# Meeting October 12

**Published:** 1886-12

**Authors:** 


*The third annual meeting of the society* was held on October 12th, at the Tremont house, F. C. Hotz, M.D., in the chair.

The following officers were elected for the ensuing year:

President, Dr. E. L. Holmes; vice-president, Dr. Lyman Ware; secretary and treasurer, Dr. Boerne Bettman.

Dr. F. C. Hotz then read a paper entitled

### THE RATIONAL TREATMENT OF PATIENTS AFTER CATARACT OPERATIONS.

Though the safety of the eye is of prime importance, we must, in our anxiety for the recovery of this organ, not entirely disregard the comfort of the patient, for we have no right to deprive our patients of any comfort which does not disturb the healing process. The usual treatment after cataract operations is anything but pleasant to the patient, and many dread the confinement to bed, and in a dark room, worse than the operation itself. If it can be shown that such measures are superfluous, they should be abandoned.

When a wound is carefully cleansed and thoroughly disinfected, and its edges are nicely approximated, it will heal by first union, provided the close adaptation of its edges is not disturbed by external violence or by muscular tractions. Rest of the wounded part is the first condition for kind healing. But to rest the eyeball it is not necessary to put the patient to bed; we can secure the necessary rest for the operated eye, i. e., we suspend the functions of the ocular muscles and immobilize the eyelids by bandaging *both* eyes. As long as they are closed they remain comparatively motionless. The bandage over both eyes accomplishes this result just as well whether the patient is in bed or sitting in a chair. The confinement to bed, therefore, is irrational, and the recumbent posture is not only of no advantage, but may even disturb the healing, because it favors the flow of blood to the head; consequently congestion of the ocular tissues is more likely to occur in the recumbent posture than when the patient is sitting up.

The uselessness of darkening the room will be apparent to everyone who will bandage his own eyes just in the same manner as we dress the eyes after cataract operations. The bandage shuts off the light so thoroughly that we will be unable to tell whether the room is light or dark. To the patient it is immaterial, but to his attendant it is a great comfort to have light in the room.

In regard to the dressing there is a difference of opinion: the majority of oculists employing the pads and bandage; some closing the eyelids only by strips of plaster, and others discarding all dressings.

If we dispense with the dark room, we cannot leave the eyes without dressing, because the winking of the eyelids and the constant rotations of the eyeball would disturb the wound and cause a good deal of irritation.

The plaster strips overcome this difficulty; but they do not give the eye any protection against mechanical insults which might cause a re-opening of the freshly united wound. The bandage with padding of absorbent cotton or any other soft material secures to the eye rest and protection against accidents and is therefore to be recommended as the most rational dressing. The flannel roller is open to the objection of being easily disarranged by the movements of the head on the pillow; but a roller of mosquito netting or Swiss gauze applied wet forms, when dry, an immovable bandage which does not become loose, and keeps nicely adjusted for any desired length of time.

The sensitiveness of the operated eye to bright light is not due to the bandage; it varies greatly in different persons; some even do not show it at all. Where it exists it only shows that the eye has not completely recovered from the effects of the operation though the external wound may appear well healed. With the eyes well bandaged my patients are sitting in easy or rocking chairs, in light rooms, and are even allowed to take an exercising walk in their rooms; they go to bed when they please, and get up whenever they like. I have never observed any complication or accident which could directly or indirectly be attributed to this mode or treatment. The recovery was as rapid and the results as good as under the old-fashioned régime.

